# A Parallel Framework for Multipoint Spiral Search in *ab Initio* Protein Structure Prediction

**DOI:** 10.1155/2014/985968

**Published:** 2014-03-16

**Authors:** Mahmood A. Rashid, Swakkhar Shatabda, M. A. Hakim Newton, Md Tamjidul Hoque, Abdul Sattar

**Affiliations:** ^1^Institute for Integrated & Intelligent Systems, Science 2 (N34) 1.45, 170 Kessels Road, Nathan, QLD 4111, Australia; ^2^Queensland Research Lab, National ICT Australia, Level 8, Y Block, 2 George Street, Brisbane, QLD 4000, Australia; ^3^Computer Science, 2000 Lakeshore Drive, Math 308, New Orleans, LA 70148, USA

## Abstract

Protein structure prediction is computationally a very challenging problem. A large number of existing search algorithms attempt to solve the problem by exploring possible structures and finding the one with the minimum free energy. However, these algorithms perform poorly on large sized proteins due to an astronomically wide search space. In this paper, we present a multipoint spiral search framework that uses parallel processing techniques to expedite exploration by starting from different points. In our approach, a set of random initial solutions are generated and distributed to different threads. We allow each thread to run for a predefined period of time. The improved solutions are stored threadwise. When the threads finish, the solutions are merged together and the duplicates are removed. A selected distinct set of solutions are then split to different threads again. In our *ab initio* protein structure prediction method, we use the three-dimensional face-centred-cubic lattice for structure-backbone mapping. We use both the low resolution hydrophobic-polar energy model and the high-resolution 20 × 20 energy model for search guiding. The experimental results show that our new parallel framework significantly improves the results obtained by the state-of-the-art single-point search approaches for both energy models on three-dimensional face-centred-cubic lattice. We also experimentally show the effectiveness of mixing energy models within parallel threads.

## 1. Introduction

Proteins are essentially linear chain of amino acids. They adopt specific folded three-dimensional structures to perform specific tasks. The function of a given protein is determined by its* native* structure, which has the lowest possible free energy level. Nevertheless, misfolded proteins cause many critical diseases such as Alzheimer's disease, Parkinson's disease, and cancer [1, 2]. Protein structures are important in drug design and biotechnology.

Protein structure prediction (PSP) is computationally a very hard problem [3]. Given a protein's amino acid sequence, the problem is to find a three-dimensional structure of the protein such that the total interaction energy amongst the amino acids in the sequence is minimised. The protein folding process that leads to such structures involves very complex molecular dynamics [4] and unknown energy factors. To deal with the complexity in a hierarchical fashion, researchers have used discretised lattice-based structures and simplified energy models [5–7] for PSP. However, the complexity of the simplified problem still remains challenging.

There are a large number of existing search algorithms that attempt to solve the PSP problem by exploring feasible structures called* conformations*. For population-based approaches, a genetic algorithm (GA^+^ [8]) reportedly produces the state-of-the-art results using hydrophobic-polar (HP) energy model. On the other hand, for local search approaches, spiral search (SS-Tabu) [9], which is a tabu-based local search, produces the best results using HP model. Both algorithms use three-dimensional (3D) face-centred-cubic (FCC) lattice for conformation representation.

The approaches used in [10–13] produced the state-of-the-art results using the high resolution Berrera 20 × 20 energy matrix (henceforth referred to as BM energy model). Nevertheless, the challenges in PSP largely remain in the fact that the energy function that needs to be minimised in order to obtain the native structure of a given protein is not clearly known. A high resolution 20 × 20 energy model (such as BM) could better capture the behaviour of the actual energy function than a low resolution energy model (such as HP). However, the fine grained details of the high resolution interaction energy matrix are often not very informative for guiding the search. Pairwise contributions that have low magnitudes could be dominated by the accumulated pairwise contributions having large magnitudes. In contrast, a low resolution energy model could effectively bias the search towards certain promising directions particularly emphasising on the pairwise contributions with large magnitudes.

In a collaborative human team, each member may work individually on his/her own way to solve a problem. They may meet together occasionally to discuss the possible ways they could find and may then refocus only on the more viable options in the next iteration. We envisage this approach to be useful in finding a suitable solution when there are enormously many alternatives that are very close to each other. We therefore try this in the context of conformational search for protein structure prediction.

In this paper, we present a multithreaded search technique that runs SS-Tabu in each thread that is guided by either HP energy or by 20 × 20 BM energy model. The search starts with a set of random initial solutions by distributing these solutions to different threads. We allow each thread to run for a predefined period of time. The interim improved solutions are stored threadwise and merged together when all threads have finished their execution. After removing the duplicates from the merged solutions, a selected distinct set of solutions is then considered for next iteration. In our approach, multipoint start first helps find some promising results. For the next set of solutions to be distributed, the most promising solutions from the merged list are selected. Therefore, multipoint parallelism reduces the search space by exploring the vicinities of the promising solutions recursively. In our parallel local search, we use both the HP energy model and 20 × 20 BM energy model on the 3D FCC lattice space. The experimental results show that our new approach significantly improves over the results obtained by the state-of-the-art single-point search approaches for the similar models.

The rest of the paper is organized as follows: Section 2 describes the background of the protein structure; Section 3 presents the related work; Section 4.1 presents the SS-Tabu algorithm used in the parallel search approach; Section 4 describes our parallel framework in detail; Section 5 discusses and analyses the experimental results; and finally, Section 6 presents the conclusions and outlines the future work.

## 2. Background

There are three computational approaches for protein structure prediction. These are* homology modeling* [14],* protein threading* [15, 16], and* ab initio* methods [17, 18]. Prediction quality of* homology modeling* and* protein threading* depends on the sequential similarity of previously known protein structures. However, our work is based on the* ab initio* approach that only depends on the amino acid sequence of the target protein. Levinthal's paradox [19] and Anfinsen's hypothesis [20] are the basis of* ab initio* methods for PSP. The idea was originated in 1970 when it was demonstrated that all information needed to fold a protein resides in its amino acid sequence. In our simplified protein structure prediction model, we use 3D FCC lattice for conformation mapping, HP and 20 × 20 BM energy models for conformation evaluation, and the spiral search algorithm [9] (SS-Tabu) in a parallel framework for conformation search. The simplified models (lattice model and energy models) and local search are described below.

### 2.1. Simplified Model

In this research, we use 3D FCC lattice points for conformation mapping to generate backbone of protein structures. We use the HP and 20 × 20 BM energy model for conformation evaluation. The 3D FCC lattice, the HP energy model, and BM energy model are briefly described below.

#### 2.1.1. 3D FCC Lattice

**Figure 1:**
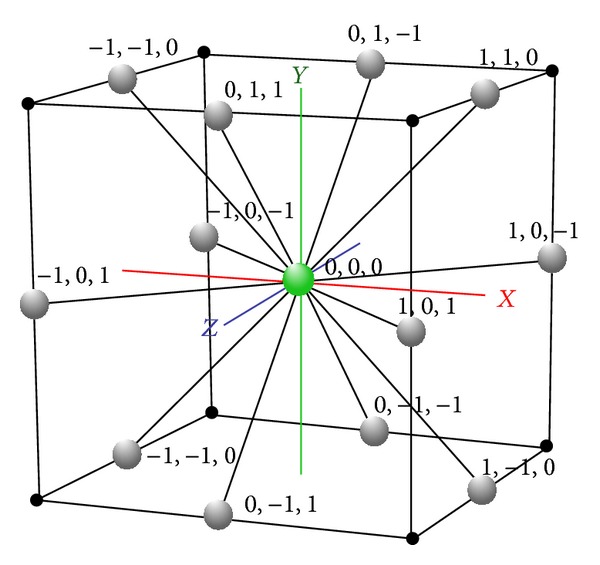
A unit 3D FCC lattice with 12 basis vectors on the Cartesian coordinates.

The FCC lattice has the highest packing density compared to the other existing lattices [21]. The hexagonal close packed (HCP) lattice, also known as cuboctahedron, was used in [22]. In HCP, each lattice point has 12 neighbours that correspond to 12 basis vertices with real-numbered coordinates, which causes the loss of structural precision for PSP. In FCC, each lattice point has 12 neighbours as shown in Figure 1.

Figure 1 shows the 12* basis vectors* with respect to the origin. The* basis vectors* are presented below denoting as $\vec{A}\cdots \vec{L}$:

(1) $$\begin{array}{c} \vec{A}=\left(1,1,0\right),\;\;\vec{B}=\left(0,1,1\right),\\ \vec{C}=\left(1,0,1\right),\;\;\vec{D}=\left(-1,1,0\right),\\ \vec{E}=\left(0,-1,1\right),\;\;\vec{F}=\left(-1,0,1\right),\\ \vec{G}=\left(1,-1,0\right),\;\;\vec{H}=\left(0,1,-1\right),\\ \vec{I}=\left(1,0,-1\right),\;\;\vec{J}=\left(-1,-1,0\right),\\ \vec{K}=\left(0,-1,-1\right),\;\;\vec{L}=\left(-1,0,-1\right). \end{array}$$

In simplified PSP, conformations are mapped on the lattice by a sequence of basis vectors or by the* relative vectors* that are relative to the previous basis vectors in the sequence.

#### 2.1.2. HP Energy Model

The 20 amino acid monomers are the building block of protein polymers. These amino acids are broadly divided into two categories based on their hydrophobicity: (a) hydrophobic amino acids (*Gly*,* Ala*,* Pro*,* Val*,* Leu*,* Ile*,* Met*,* Phe*,* Tyr*,* Trp*) denoted by H; and (b) hydrophilic or polar amino acids (*Ser*,* Thr*,* Cys*,* Asn*,* Gln*,* Lys*,* His*,* Arg*,* Asp*,* Glu*) denoted by P. In the HP model [23], when two nonconsecutive hydrophobic amino acids become topologically neighbours, they contribute a certain amount of negative energy, which for simplicity is shown as −1 in Table 1. The total energy (*E*) of a conformation based on the HP model becomes the sum of the contributions of all pairs of nonconsecutive hydrophobic amino acids as follows:

(2) $$\begin{array}{c} E=\underset{i<j-1}{\sum }c_{ij}\cdot e_{ij}. \end{array}$$

Here, *c*_*ij*_ = 1 if amino acids *i* and *j* are nonconsecutive neighbours on the lattice, otherwise 0; and *e*_*ij*_ = −1 if *i*th and *j*th amino acids are hydrophobic, otherwise 0.

### 2.2. BM Energy Model

By analysing crystallised protein structures, Miyazawa and Jernigan [24] in 1985 statistically deduced a 20 × 20 energy matrix that considers residue contact propensities between the amino acids. By calculating empirical contact energies on the basis of information available from selected protein structures and following the quasichemical approximation Berrera et al. [25] in 2003 deduced another 20 × 20 energy matrix. In this work, we use the latter model and denote it by BM energy model.  Table 2 shows the BM energy model with amino acid names at the left-most column and the bottom-most row and the interaction energy values in the cells. The amino acid names that have boldface are hydrophobic. We draw lines in Table 2 to show groupings based on H-H, H-P, and P-P interactions. In the context of this work, it is worth noting that most energy contributions that have large magnitudes are from H-H interactions followed by those from H-P interactions.

The total energy *E*_bm_ (shown in (3)) of a conformation based on the BM energy model is the sum of the contributions over all pairs of nonconsecutive amino acids that are one unit lattice distance apart:

(3) $$\begin{array}{c} E_{\text{bm}}=\underset{i<j-1}{\sum }c_{ij}\cdot e_{ij}. \end{array}$$

Here, *c*_*ij*_ = 1 if amino acids at positions *i* and *j* in the sequence are nonconsecutive neighbours on the lattice, otherwise 0; and *e*_*ij*_ is the empirical energy value between the *i*th and *j*th amino acid pair specified in the matrix for the BM model.

### 2.3. Local Search

Starting from an initial solution, local search algorithms move from one solution to another to find a better solution. Local search algorithms are well known for efficiently producing high quality solutions [9, 26, 27], which are difficult for systematic search approaches. However, they are incomplete [28] and suffer from revisitation and stagnation. Restarting the whole or parts of a solution remains the typical approach to deal with such situations.

### 2.4. Tabu Metaheuristic

Tabu metaheuristic [29, 30] enhances the performance of local search algorithms. It maintains a short-term memory storage to remember the local changes of a solution. Then any further local changes for those stored positions are forbidden for a certain number of subsequent iterations (known as tabu tenure).

## 3. Related Work

There are a large number of existing search algorithms that attempt to solve the PSP problem by exploring feasible structures on different energy models. In this section we explore the works related to HP and 20 × 20 energy models as below.

### 3.1. HP Energy-Based Approaches

Different types of metaheuristic have been used in solving the simplified PSP problem. These include Monte Carlo Simulation [31], Simulated Annealing [32], Genetic Algorithms (GA) [33, 34], Tabu Search with GA [35], Tabu Search with Hill Climbing [36], Ant Colony Optimisation [37], Immune Algorithms [38], Tabu-based Stochastic Local Search [26, 27], and Constraint Programming [39].

The Bioinformatics Group, headed by Rolf Backofen, applied Constraint Programming [40–42] using exact and complete algorithms. Their exact and complete algorithms work efficiently if similar hydrophobic core exists in the repository.

Cebrián et al. [26] used tabu-based local search, and Shatabda et al. [27] used memory-based local search with tabu heuristic and achieved the state-of-the-art results. However, Dotu et al. [39] used constraint programming and found promising results but only for smaller sized (length < 100 amino acids) proteins. Besides local search, Unger and Moult [33] applied population-based genetic algorithms to PSP and found their method to be more promising than the Monte Carlo-based methods [31]. They used absolute encodings on the square and cubic lattices for HP energy model. Later, Patton [43] used relative encodings to represent conformations and a penalty method to enforce the self-avoiding walk constraint. GAs have been used by Hoque et al. [22] for cubic and 3D HCP lattices. They used DFS-generated pathways [44] in GA crossover for protein structure prediction. They also introduced a twin-removal operator [45] to remove duplicates from the population to prevent the search from stalling. Ullah et al. in [12, 46] combined local search with constraint programming. They used a 20 × 20 energy model [25] on FCC lattice and found promising results. In another hybrid approach [47], tabu metaheuristic was combined with genetic algorithms in two-dimensional HP model to observe crossover and mutation rates over time.

However, for the simplified model (HP energy model and 3D FCC lattice) that is used in this paper, a new genetic algorithm GA^+^ [8] and a tabu-based local search algorithm Spiral Search [9] currently produce the state-of-the-art results.

### 3.2. Empirical 20 × 20 Matrix Energy Based Approaches

A constraint programming technique was used in [48] by Dal Palù et al. to predict tertiary structures of real proteins using secondary structure information. They also used constraint programming with different heuristics in [49] and a constraint solver named COLA [50] that is highly optimized for protein structure prediction. In another work [51], a fragment assembly method was utilised with empirical energy potentials to optimise protein structures. Among other successful approaches, a population-based local search [52] and a population-based genetic algorithm [13] were used with empirical energy functions.

In a hybrid approach, Ullah and Steinöfel [12] applied a constraint programming-based large neighbourhood search technique on top of the output of COLA solver. The hybrid approach produced the state-of-the-art results for several small sized (less than 75 amino acids) benchmark proteins.

In another work, Ullah et al. [46] proposed a two stage optimisation approach combining constraint programming and local search. The first stage of the approach produced compact optimal structures by using the CPSP tools based on the HP model. In the second stage, those compact structures were used as the input of a simulated annealing-based local search that is guided by the BM energy model.

In a recent work [10], Shatabda et al. presented a mixed heuristic local search algorithm for PSP and produced the state-of-the-art results using BM energy model on 3D FCC lattice. The mixed heuristic local search in each iteration randomly selects a heuristic from a given number of heuristics designed by the authors. The selected heuristics are then used in evaluating the generated neighbouring solutions of the current solution. Although the heuristics themselves are weaker than the BM energy, their collective use in the random mixing fashion produces results better than the BM energy itself.

### 3.3. Parallel Approaches

Vargas and Lopes [53] proposed an Artificial Bee Colony algorithm based on two parallel approaches (master slave and a hybrid hierarchical) for protein structure prediction using the 3D HP model with sidechains. They showed that the parallel methods achieved a good level of efficiency while compared with the sequential version. A comparative study of parallel metaheuristics was conducted by Trantar et al. [54] using a genetic algorithm, a simulated annealing algorithm, and a random search method in grid environments for protein structure prediction. In another work [55], they applied a parallel hybrid genetic algorithm in order to efficiently deal with the PSP problem using the computational grid. They experimentally showed the effectiveness of a computational grid-based approach. All-atom force field-based protein structure prediction using parallel particle swarm optimization approach was proposed by Kandov in [56]. He showed that asynchronous parallelisation speeds up the simulation better than the synchronous one and reduces the effective time for predictions significantly. Among others, Calvo et al. in [57, 58] applied a parallel multiobjective evolutionary approach and found linear speedups in structure prediction for benchmark proteins and Robles et al. in [59] applied parallel approach in local search to predict secondary structure of a protein from its amino acid sequence.

## 4. Our Approach

The driving force of our parallel search framework is SS-Tabu [9] that has two versions: (i) the existing algorithm, designed for HP model (as shown in Algorithm 1 and described in Section 4.1) and (ii) the customised spiral search algorithm, designed for 20 × 20 BM energy model (as shown in Algorithm 5 and described in Section 4.2). We feed the two versions of spiral search algorithms in different threads in different combinations. The variations are described in the experimental results section.

### 4.1. SS-Tabu: Spiral Search

SS-Tabu is a hydrophobic core directed local search [9] that works in a spiral fashion. This algorithm (the* pseudocode* in Algorithm 1) is the basis of the proposed parallel local search framework. SS-Tabu is composed of H and P move selections, random-walk [60], and relay-restart [9]. However, this algorithm is further customised for detailed 20 × 20 energy model as described in Section 4.2. Both versions of SS-Tabu are used in parallel threads with different combinations within the parallel framework. The features of existing SS-Tabu are described in Algorithm 1.

#### 4.1.1. Applying Diagonal Move

**Figure 2:**
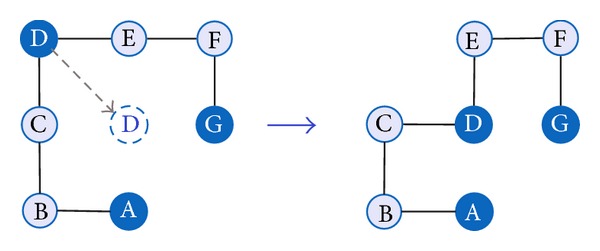
Diagonal move operator. For easy understanding, the figures are presented in 2D space.

In a tabu-guided local search (see Algorithm 1), we use the diagonal move operator (shown in Figure 2) to build H-core. A diagonal move displaces *i*th amino acid from its position to another position on the lattice without changing the position of its succeeding (*i* + 1)th and preceding (*i* − 1)th amino acids in the sequence. The move is just a corner-flip to an unoccupied lattice point.

#### 4.1.2. Forming H-Core

**Figure 3:**
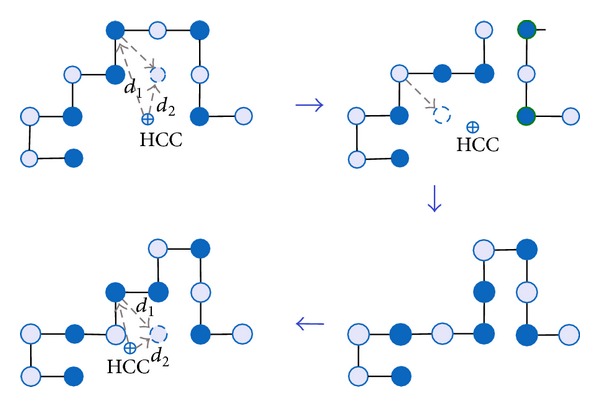
Spiral search comprising a series of diagonal moves with tabu metaheuristics. For simplification and easy understanding, the figures are presented in 2D space.

Protein structures have hydrophobic cores (H-core) that hide the hydrophobic amino acids from water and expose the polar amino acids to the surface to be in contact with the surrounding water molecules [61]. H-core formation is an important objective for HP-based protein structure prediction models. In our work, we repeatedly use the diagonal-move to aid forming the H-core. We maintain a tabu list to control the amino acids from getting involved in the diagonal moves. SS-Tabu performs a series of diagonal moves on a given conformation to build the H-core around the hydrophobic core centre (HCC) as shown in Figure 3. The Cartesian distance between the HCC and the current position or a new position is denoted by *d*_1_ and *d*_2_, respectively. The diagonal move squeezes the conformation and quickly forms the H-core in a spiral fashion.

#### 4.1.3. Selecting Moves for HP Model

In H-move selection algorithm (Algorithm 2), the HCC is calculated (Line 2) by finding arithmetic means of *x*, *y*, and *z* coordinates of all hydrophobic amino acids using (4). The selection is guided by the Cartesian distance *d*_*i*_ (as shown in (5)) between HCC and the hydrophobic amino acids in the sequence. For the *i*th hydrophobic amino acid, the common topological neighbours of the (*i* − 1)th and (*i* + 1)th amino acids are computed. The topological neighbours (TN) of a lattice point are the points at unit lattice-distance apart from it. From the common neighbours, the unoccupied points are identified. The Cartesian distance of all unoccupied common neighbours is calculated from the HCC using (5). Then the point with the shortest distance is picked. This point is listed in the possible H-move list for *i*th hydrophobic amino acid if its current distance from HCC is greater than that of the selected point. When all hydrophobic amino acids are traversed and the feasible shortest distances are listed in H-move list, the amino acid having the shortest distance in H-move list is chosen to apply the diagonal move on it (Algorithm 1 Line 9). A tabu list is maintained for each hydrophobic amino acid to control the selection priority amongst them. For each successful move, the tabu list is updated for the respective amino acid. The process stops when no H-move is found. In this situation, the control is transferred to select and apply P-moves. Consider

(4) $$\begin{array}{c} x_{\text{hcc}}=\frac{1}{n_{h}}\overset{n_{h}}{\underset{i=1}{\sum }}x_{i},\;\;y_{\text{hcc}}=\frac{1}{n_{h}}\overset{n_{h}}{\underset{i=1}{\sum }}y_{i},\;\;z_{\text{hcc}}=\frac{1}{n_{h}}\overset{n_{h}}{\underset{i=1}{\sum }}z_{i}, \end{array}$$

where *n*_*h*_ is the number of H amino acids in the protein. Consider

(5) $$\begin{array}{c} d_{i}=\sqrt{{\left(x_{i}-x_{\text{hcc}}\right)}^{2}+{\left(y_{i}-y_{\text{hcc}}\right)}^{2}+{\left(z_{i}-z_{\text{hcc}}\right)}^{2}}. \end{array}$$

However, in P-move selection (Algorithm 1 Line 12), the same kind of diagonal moves is applied as H-move. For each *i*th polar amino acid, all free lattice points that are common neighbours of lattice points occupied by (*i* − 1)th and (*i* + 1)th amino acids are listed. From the list, a point is selected randomly to complete a diagonal move (Algorithm 1, Line 14) for the respective polar amino acid. No hydrophobic-core-center is calculated, no Cartesian distance is measured, and no tabu list is maintained for P-move. After one try for each polar amino acid the control is returned to select and apply H-moves.

#### 4.1.4. Handling Stagnation

For hard optimisation problems such as protein structure prediction, local search algorithms often face stagnation. In HP model-based conformational search, stagnation is encountered when a premature H-core is formed. Handling the stagnations is a challenging issue for conformational search algorithms (e.g., GA, LS). Thus, handling such situation intelligently is important to proceed further. To deal with stagnation, in SS-Tabu, random-walk [60] and relay-restart techniques are used on an on-demand basis.

*Random-Walk.* Premature H-cores are observed at local minima. To escape local minima, a random-walk [60] algorithm (Algorithm 1, Line 27) is applied. This algorithm uses pull moves [62] to break the premature H-cores and to create diversity.

*Relay-Restart.* When the search stagnation situation arises, a new relay-restart technique (Algorithm 1 Line 23) is applied instead of a fresh restart or restarting from the current best solution [26, 27]. We use relay-restart when random-walk fails to escape from the local minima. The relay-restart starts from an improving solution. We maintain an improving solution list that contains all the improving solutions after the initialisation.

#### 4.1.5. Further Implementation Details

Like other search algorithms, SS-Tabu requires initialisation. It also needs evaluation of the solution in each iteration. It starts with a randomly generated or parameterised initial solution and enhances it in a spiral fashion. Further, it needs to maintain a tabu meta-heuristic to guide the local search. 

*Tabu Tenure.* Intuitively we use different tabu-tenure values based on the number of hydrophobic amino acids (hCount) in the sequence. We calculate tabu-tenure using the following formula:

(6) $$\begin{array}{c} \text{tenure}=\left(10+\frac{h\text{Count}}{10}\right). \end{array}$$

The tabu-tenure calculated using (6) is used at Lines 5, 24, and 28 in Algorithm 1 during initialising and resetting tabu-list.

*Evaluation.* After each iteration, the conformation is evaluated by counting the H-H contacts (topological neighbours) where the two amino acids are nonconsecutive. The* pseudocode* in Algorithm 3 presents the algorithm of calculating the free energy of a given conformation. Note that the energy value is negation of the H-H contact count. For 20 × 20 BM energy model the pairwise contact potentials are found in matrix presented in Table 2. 

*Initialisation.* Our algorithm starts with a feasible set of conformations known as population. We generate each initial conformation following a randomly generated self-avoiding walk (SAW) on FCC lattice points. The* pseudocode* of the algorithm is presented in Algorithm 4. It places the first amino acid at (0,0, 0). It then randomly selects a basis vector to place the successive amino acid at a neighbouring free lattice point. The mapping proceeds until a self-avoiding walk is found for the whole protein sequence.

### 4.2. BM Model Adopted Spiral Search

The basic difference between the HP energy based original spiral search (SS-Tabu [9]) and the BM energy guided adopted spiral search lies on the move selection criteria. In former version of spiral search, the amino acids are divided into two groups (H and P). The moves are selected based on these two properties of the amino acids that are guided by the distance of H amino acid from the HCC. However, to adopt 20 × 20 BM energy model, all 20 amino acids need to be taken into consideration and the move selection criteria are guided by the distance of any amino acid from the core centre (CC) of the current structure (Algorithm 5). The CC and the distance are calculated using (7) and (8), respectively.

#### 4.2.1. Selecting Moves for BM (20 × 20) Model

In move selection (Algorithm 5 Line 6), the CC is calculated by finding arithmetic means of *x*, *y*, and *z* coordinates of all amino acids using (7). The selection is guided by the Cartesian distance *d*_*i*_ (as shown in (5)) between CC and the amino acids in the sequence. For the *i*th amino acid, the common topological neighbours of the (*i* − 1)th and (*i* + 1)th amino acids are computed. The topological neighbours (TN) of a lattice point are the points at unit lattice distance apart from it. From the common neighbours, the unoccupied points are identified. The Cartesian distance of all unoccupied common neighbours is calculated from the CC using (8). Then the point with the shortest distance is picked. This point is listed in the possible move list for *i*th amino acid if its current distance from CC is greater than that of the selected point. When all amino acids are traversed and the feasible shortest distances are listed in move list, the amino acid having the shortest distance in move list is chosen to apply the diagonal move on it (Algorithm 5, Line 8). A tabu list is maintained for each amino acid to control the selection priority amongst them. For each successful move, the tabu list is updated (Algorithm 5, Line 9) for the respective amino acid:

(7) $$\begin{array}{c} x_{\text{cc}}=\frac{1}{n}\overset{n}{\underset{i=1}{\sum }}x_{i},\;\;y_{\text{cc}}=\frac{1}{n}\overset{n}{\underset{i=1}{\sum }}y_{i},\;\;z_{\text{cc}}=\frac{1}{n}\overset{n}{\underset{i=1}{\sum }}z_{i}, \end{array}$$

where *n* is the number of amino acids in the protein. Consider

(8) $$\begin{array}{c} d_{i}=\sqrt{{\left(x_{i}-x_{\text{cc}}\right)}^{2}+{\left(y_{i}-y_{\text{cc}}\right)}^{2}+{\left(z_{i}-z_{\text{cc}}\right)}^{2}}. \end{array}$$

### 4.3. Parallel Framework

In our implemented prototype, we use four parallel threads. The two versions of SS-Tabu are distributed amongst the four threads as shown in Table 3.

**Figure 4:**
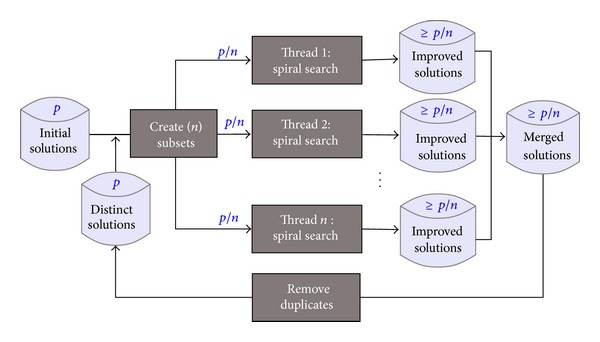
Parallel spiral search framework.

Figure 4 shows the architecture of our parallel search algorithm. In this framework, the search starts with a set of randomly generated initial solutions (Line 2 in Algorithm 6). The solutions are then divided in subsets (Line 4 in Algorithm 6) and are distributed to different threads.

We allow each thread to run for a predefined period of time. The improved solutions are stored threadwise and are merged together (Line 9 in Algorithm 6) when all threads finish. After removing the duplicates (Line 10 in Algorithm 6) from the merged solutions, a selected distinct set of solutions are taken (Line 11 in Algorithm 6) for the next iteration. The iterative process continues until the terminating criteria (Line 3 in Algorithm 6) are satisfied.

## 5. Experimental Results and Analyses

We conduct our experiments on two different sets of benchmark proteins: HP benchmarks and 20 × 20 benchmarks. The rest of this section will present the experimental results in detail.

### 5.1. Experiment Setup

#### 5.1.1. Implementation

The parallel spiral search framework has been implemented in Java 6.0 using Java standard APIs. Currently the source code is not available publicly due to the legal bindings. However, an executable version of the application could be requested to the corresponding author.

#### 5.1.2. Execution

We ran our experiments on the NICTA (NICTA website: http://www.nicta.com.au/) cluster. The cluster consists of a number of identical Dell PowerEdge R415 computers, each equipped with 2 × AMD 6-Core Opteron 4184 processors, 2.8 GHz clock speed, 3M L2/6M L3 Cache, 64 GB memory, and running Rocks OS (a Linux variant for cluster). The experimental results presented in this paper are obtained from 50 different runs of identical settings for each protein when using HP benchmarks and 20 different runs of identical settings for each protein when using 20 × 20 benchmarks.

### 5.2. Experimental Results on HP Benchmark

The experimental results on HP benchmarks are presented in Tables 4 and 5. Amongst the sequences, F90, S, F180, and R instances are taken from Peter Clote laboratory website (Peter Clote Lab: http://bioinformatics.bc.edu/clotelab/FCCproteinStructure/). These instances have been used in [8, 9, 26, 27, 39] for evaluating different algorithms. Moreover, we use other six larger sequences that are taken from the CASP (CASP website: http://predictioncenter.org/casp9/targetlist.cgi) competition. The corresponding CASP target IDs for proteins* 3mse*,* 3mr7*,* 3mqz*,* 3no6*,* 3no3*, and* 3on7* are* T0521*,* T0520*,* T0525*,* T0516*,* T0570*, and* T0563*. These CASP targets are also used in [27]. To fit in the HP model, the CASP targets are converted to HP sequences based on the hydrophobic properties of the constituent amino acids. The lower bounds of the free energy values (in Column LBFEof Tables 4 and 5) are obtained from [26, 27]; however, there are some unknown values (presented as *n*/*a*) of lower bounds of free energy for large sequences.

#### 5.2.1. Results on Medium Sized HP Benchmark Proteins

In Table 4, we present three different sets of result obtained from (i) our parallel local search framework that runs on four parallel threads (30 minutes/run), (ii) a local search (SS-Tabu) that runs on a single thread (2 hours/run), and (iii) a genetic algorithm (GA^+^) that runs on a single thread (2 hours/run). In the table, the* Size* column presents the number of amino acids in the sequences, and the *LBFE* column shows the known lower bounds of free energy for the corresponding protein sequences in Column *ID*. The best and average free energy values for three different algorithms are presented in the table under the specific column headers (PSS, SS-Tabu, and GA^+^). The RI Columns present the relative improvements of parallel local search over the single-thread local search and the genetic algorithm. The bold-faced values indicate better performance in comparison to the other algorithms for corresponding proteins.

#### 5.2.2. Results on Large Sized HP Benchmark Proteins

In Table 5, we present three different sets of result obtained from (i) our parallel local search framework that runs on four parallel threads (1 hour 15 minutes/run), (ii) a local search (SS-Tabu) that runs on a single thread (5 hours/run), and (iii) a genetic algorithm (GA^+^) that runs on a single thread (5 hours/run). In the table, the* Size* column presents the number of amino acids in the sequences, and the LBFEcolumn shows the known lower bounds of free energy for the corresponding protein sequences in Column* ID*. However, a lower bound of free energy for protein* 3on7* is not known. The best and average free energy values for three different algorithms are presented in the table under the specific column headers (PSS, SS-Tabu, and GA^+^). The RI Columns present the relative improvements of parallel local search over the single-thread local search and the genetic algorithm. The bold-faced values indicate better performance in comparison to the other algorithms for corresponding proteins.

#### 5.2.3. Relative Improvement on HP Benchmark

The difficulty of improving energy level is increased as the improved energy level approaches to the lower bound of free energy. For example, if the lower bound of free energy of a protein is −100, the efforts to improve energy level from −80 to −85 are much less than that to improve energy level from −95 to −100 though the change in energy is the same (−5). Relative Improvement (RI) explains how close our predicted results are to the lower bound of free energy with respect to the energy obtained from the state-of-the-art approaches:

(9) $$\begin{array}{c} \text{RI}=\frac{E_{t}-E_{r}}{E_{l}-E_{r}}*100\%. \end{array}$$

In Tables 4 and 5, we also present a comparison of improvements (%) on average conformation quality (in terms of free energy levels). We compare PSS (target) with SS-Tabu and GA^+^ (references). For each protein, the RI of the target (*t*) with respect to the reference (*r*) is calculated using the formula in (9), where *E*_*t*_ and *E*_*r*_ denote the average energy values achieved by the target and the reference, respectively, and *E*_*l*_ is the lower bound of free energy for the protein in the HP model. We present the relative improvements only for the proteins having known lower bounds of free energy values. We test our new approach on 16 different proteins of various lengths. The bold-faced values are the minimum and the maximum improvements for the same column.

*Improvement with respect to SS-Tabu.* The experimental results in Tables 4 and 5, at column RI under SS-Tabu, show that our PSS is able to improve the search quality in terms of minimising the free energy level over all the 16 proteins considered for the test. The relative improvements with respect to SS-Tabu range from 0% to 50%.

*Improvement with respect to G* *A* ^+^. The experimental results in Tables 4 and 5, at column RI (relative improvement) under GA^+^, show that our PSS is able to improve the search quality in terms of minimising the free energy level over all 16 proteins considered for the test. The relative improvements with respect to GA^+^ range from 0% to 33%.

#### 5.2.4. Search Progress

**Figure 5:**
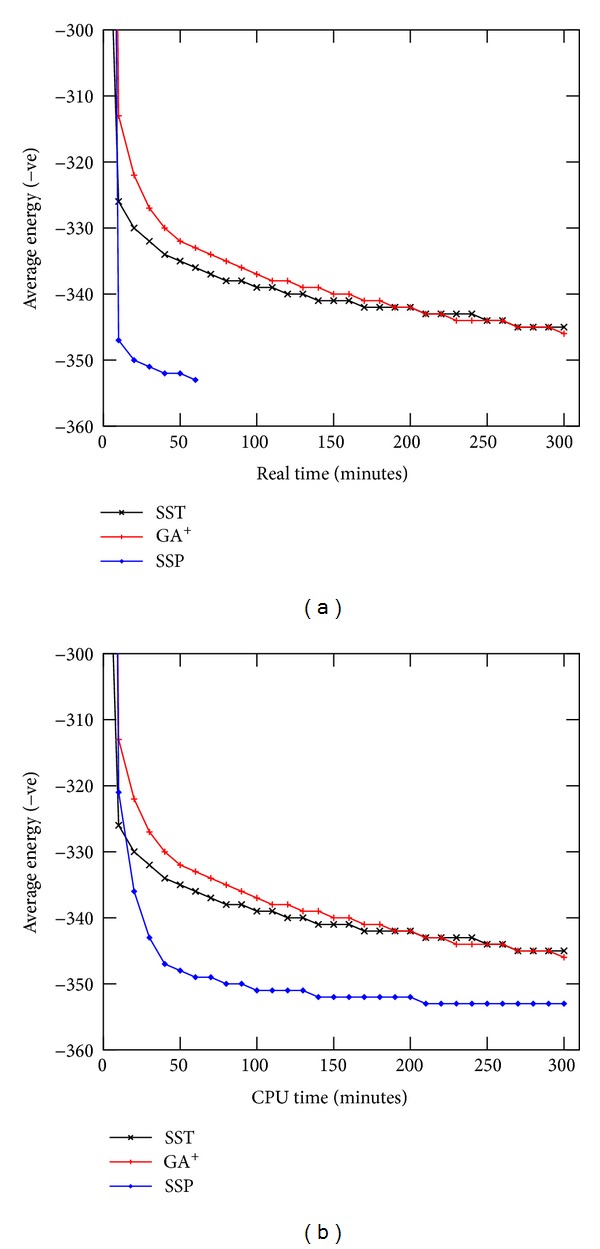
Search progress for protein R1 with (a) real time and (b) CPU time of 4 threads (4x real time). SST, GA^+^, and SSP represent tabu-based spiral search [9], genetic algorithms [8], and multipoint parallel spiral search, respectively.

We compare the search progresses of SS-Tabu, GA^+^, and PSS on the basis of real execution time.  Figure 5(a) shows the average energy values obtained with times by the algorithms for protein R1. The graph shows that the progress of PSS stops at 75 minutes (1.25 hours). As we mentioned earlier, we run parallel threads (four threads) in our PSS for 1.25 hours to keep total CPU time equal to five (1.25 × 4 = 5) hours. From the graph, it is clear that multipoint local search with four parallel threads dramatically outperforms the local search and genetic algorithms within (1/4)th of the execution time.

However, in Figure 5(b), we compare the search progresses of SS-Tabu, GA^+^, and PSS over CPU time. The CPU time of PSS is calculated by summing up the individual times of all threads (time per thread × 4) in different instances.

#### 5.2.5. Comments on Our HP-Based Method

In Tables 4 and 5, the Columns LBFE represent the lower bound of free energy. Some of these values are taken from the literatures and others are obtained running exact and complete algorithms based CPSP-tools [42]. However, we do not compare our experimental results with results obtained from CPSP tools because of a fundamental conceptual difference between our approaches and Will and Backofen [63, 64]. Will's HPstruct algorithm [65] proceeds with threading an input HP sequence onto hydrophobic cores from a collection of precomputed and stored H-cores. On the other hand, our algorithms compute H-cores on the fly like Yue-Dill CHCC method [61, 66]. HPstruct requires a precomputed set of H-cores for the number of H amino acids in the given sequence. Therefore, CPSP tools cannot find structure without the availability of a precomputed optimal H-core.

### 5.3. Experimental Results on 20 × 20 Benchmark

Besides HP energy model, we apply our parallel framework on standard 20 × 20 benchmark proteins. The protein instances used in our experiments are taken from the literature (as shown in Table 6). The first seven proteins* 4RXN*,* 1ENH*,* 4PTI*,* 2IGD*,* 1YPA*,* 1R69*, and* 1CTF* are taken from [12] and the next five proteins* 3MX7*,* 3NBM*,* CMQO*,* 3MRO*, and* 3PNX *from [10]. In Table 7, we present eight sets of experimental results. The approaches are described below.

1. * LS-Tabu* is heuristically guided local search based on tabu metaheuristic. The result presented in Table 7 under Column LS-Tabu is the output of 20 different runs of LS-Tabu [10] in an identical setting over 60 minutes duration. The algorithm runs on a single thread using Berrera et al. 20 × 20 energy model.
2. * SS-Tabu* is core directed local search based on tabu metaheuristic works in an spiral fashion. The result presented in Table 7 under Column SS-Tabu is the output of 20 different runs of LS-Tabu [9] in an identical setting over 60 minutes duration. The algorithm runs on a single thread using Berrera et al. 20 × 20 energy model.
3. * PSSB4H0* is a variant of parallel spiral search running in 4 threads. In this variant of PSS, in all 4 threads, the SS-Tabu is guided by Berrera et al. 20 × 20 energy model. The parallel threads are terminated after 15 minutes. Therefore, the total CPU time remains (15 × 4-threads) the same as the SS-Tabu or LS-Tabu.
4. * PSSB3H1* is a variant of parallel spiral search running in 4 threads. In this variant of PSS, in 3 threads, the SS-Tabu is guided by Berrera et al. 20 × 20 energy model and in other threads, the SS-Tabu is guided by HP energy model. The parallel threads are terminated after 15 minutes. Therefore, the total CPU time remains (15 × 4-threads) the same as the SS-Tabu or LS-Tabu.
5. * PSSB2H2* is a variant of parallel spiral search running in 4 threads. In this variant of PSS, in 3 threads, the SS-Tabu is guided by Berrera et al. 20 × 20 energy model and in other 2 threads, the SS-Tabu is guided by HP energy model. The parallel threads are terminated after 15 minutes. Therefore, the total CPU time remains (15 × 4-threads) the same as the SS-Tabu or LS-Tabu.
6. * PSSB1H3* is a variant of parallel spiral search running in 4 threads. In this variant of PSS, in 3 threads, the SS-Tabu is guided by Berrera et al. 20 × 20 energy model and in other 3 threads, the SS-Tabu is guided by HP energy model. The parallel threads are terminated after 15 minutes. Therefore, the total CPU time remains (15 × 4-threads) the same as the SS-Tabu or LS-Tabu.
7. * PSSB0H4* is a variant of parallel spiral search running in 4 threads. In this variant of PSS, in all 4 threads, the SS-Tabu is guided by HP energy model. The parallel threads are terminated after 15 minutes. Therefore, the total CPU time remains (15 × 4-threads) the same as the SS-Tabu or LS-Tabu.
8. *GA* ^+^ is population-based genetic algorithm that uses hydrophobic-core directed macromutation operator and random-walk-based stagnation recovery technique in addition to the regular GA operators. The result presented in Table 7 under Column GA^+^ is the output of 20 different runs of GA^+^ [11] in an identical setting over 60 minutes duration. The algorithm runs on a single thread using both HP and BM energy models in a mixing manner.

### 5.4. Energy Values on 20 × 20 Benchmark

In Table 7, the energy columns show the energy values obtained from different approaches on 12 benchmark proteins (Table 6). Although the searches are guided by both HP and BM energy models, the energy values are calculated by applying Berrera et al. 20 × 20 energy matrix. The experimental results show that amongst the parallel spiral search variants, PSSB1H3 (6 out of 12 proteins) and PSSB0H4 (6 out of 12 proteins) produce better results in comparison to the other variants in terms of lowest interaction energies. However, the GA^+^ performs better in comparison to the parallel spiral search variants for 9 out of 12 proteins.

### 5.5. RMSD Values on 20 × 20 Benchmark

The RMSD is frequently used to measure the differences between values predicted by a model and the values actually observed. We compare the predicted structures obtained by our approach with the state-of-the-art approaches by measuring the RMSD with respect to the native structures from PDB. For any given structure, the RMSD is calculated using (10). The average distance between two *α*-Carbons in a native structure is 3.8 Å. To calculate RMSD, the distance between two neighbour lattice points ($\sqrt{2}$ for FCC lattice) is considered as 3.8 Å. Consider

(10) $$\begin{array}{c} \text{RMSD}=\sqrt{\frac{\sum_{i=1}^{n-1}\sum_{j=i+1}^{n}{\left(d_{ij}^{p}-d_{ij}^{n}\right)}^{2}}{n*\left(n-1\right)/2}}, \end{array}$$

where *d*_*ij*_^*p*^ and *d*_*ij*_^*n*^ denote the distances between *i*th and *j*th amino acids, respectively, in the predicted structure and the native structure of the protein.

In Table 7, the RMSD columns show the root-mean-square deviation (RMSD) values obtained from different approaches on 12 benchmark proteins (Table 6). The experimental results show that amongst the parallel spiral search variants, PSSB1H3 (7 out of 12 proteins) produces better results in comparison to other variants in terms of lowest RMSD values. However, when compared with GA^+^, the parallel variants perform better for 11 out of 12 proteins.

### 5.6. Effect of Mixing Energy Models

The best hydrophobic cores do not always correspond to the best structures in terms of RMSD values [67, 68]. These observations inspired us to mix the energy models. The approaches presented in Table 7 are guided by BM, HP, or both energy models. However, the conformations are always evaluated using BM model. The experimental results show that when the variants are guided by HP or both BM and HP models (such as PSSB3H1, PSSB2H2, PSSB1H3, and PSSB0H4) it performs better than the variant guided by BM model (such as PSSB4H0). Therefore, from the observation of RMSD values, it is clear that HP model works as a better guidance heuristic, whereas BM model works as better model for evaluating conformations.

## 6. Conclusion

In this paper, we present a multipoint parallel local search framework that runs tabu-based local search (spiral search [9]) in parallel threads. In our* ab initio* protein structure prediction method, we develop two versions of SS-Tabu that uses hydrophobic-polar energy model and 20 × 20 Berrera et al. [25] energy model separately on face-centred-cubic lattice. Collaboration and negotiation play vital roles in dealing with real world challenges. In our research, we try to adopt this analogy by considering each thread as a collaborator. We allow each thread to run for a predefined period of time. The threads are met in an assembly point when they finish their execution and donate or accept better solutions to proceed with. The PSS starts with a set of random initial solutions by distributing a subset of solutions to different threads which are running different combinations of two versions of SS-Tabu. The interim improved solutions are stored threadwise and merged together when the threads finish. After removing the duplicates from the merged solutions, a selected distinct set of solutions is considered for the next iteration. In our approach, multipoint start helps find some promising solutions. For the next working set of solutions from the merged list, the most promising solutions are selected. Therefore, multipoint parallelism reduces the search space by exploring around the promising solutions in every iteration. The experimental results show that our new approach significantly improves over the results obtained by the state-of-the-art single-point search approaches.

## Figures and Tables

**Algorithm 1:**
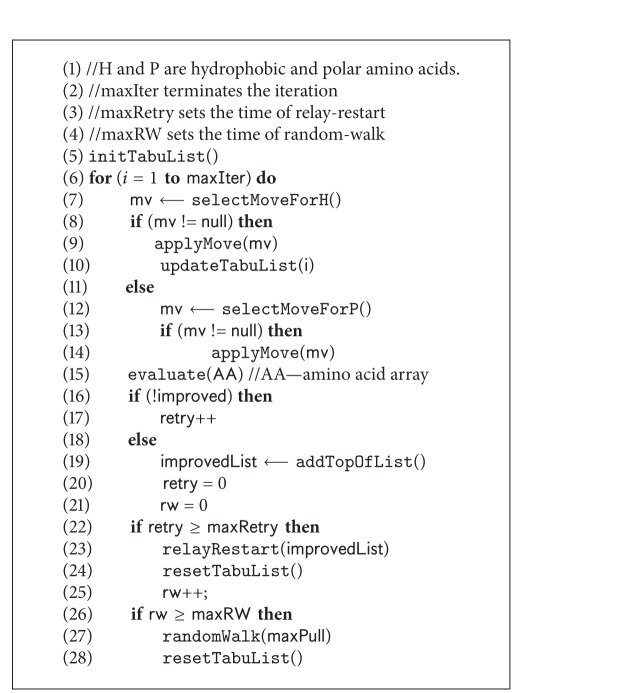
*SpiralSearchHP*(**C**).

**Algorithm 2:**
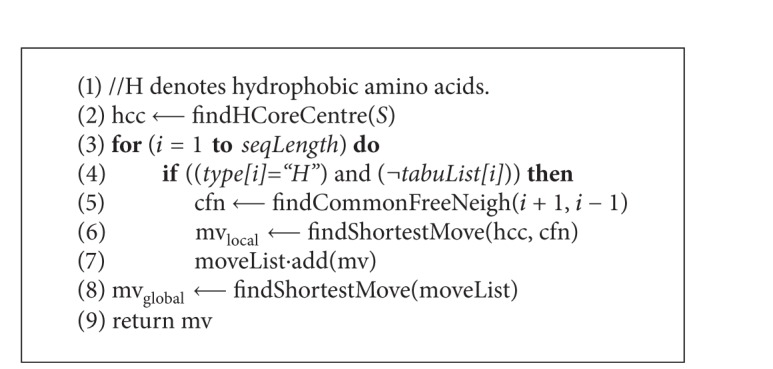
The *pseudocode *of H-move selection: selectMoveForH().

**Algorithm 3:**
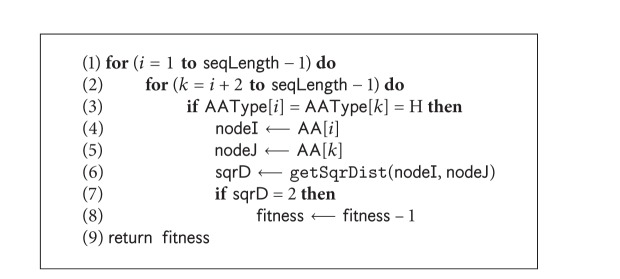
*evaluate*(**AA**).

**Algorithm 4:**
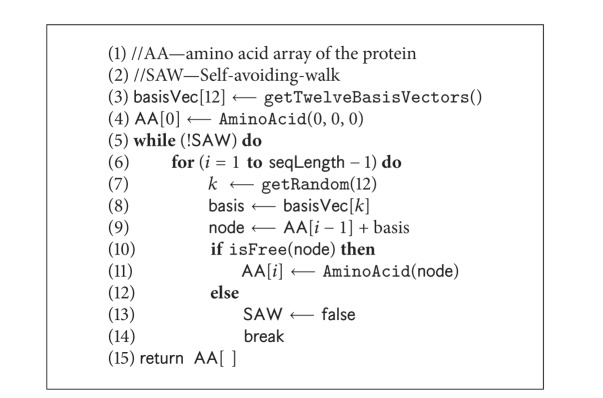
*initialise*().

**Algorithm 5:**
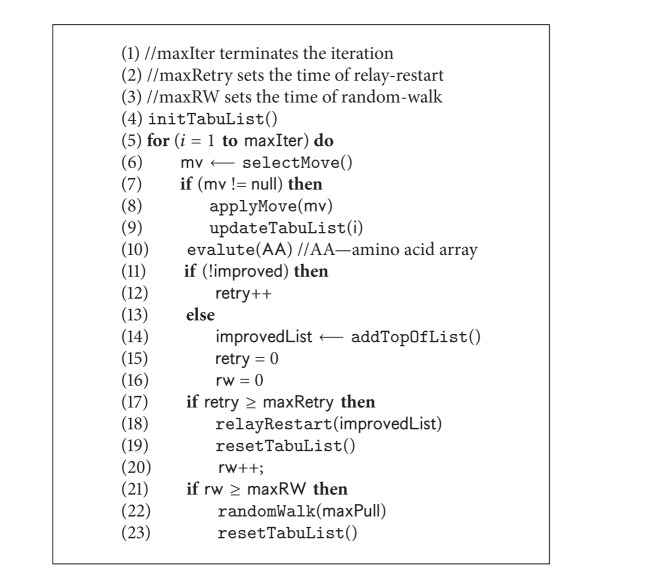
*SpiralSearchBM*(**C**).

**Algorithm 6:**
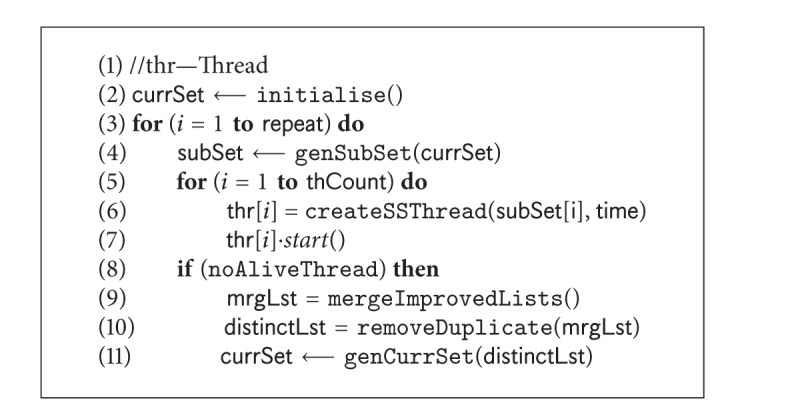
*SSParallel*(**time**, **repeat**).

**Table 1:** HP energy model [23].

	H	P
H	−1	0
P	0	0

**Table 2:** The 20 × 20 BM energy model by Berrera et al. [25].

*Cys *	−3.477																			
**Met**	−2.24	−1.901																		
**Phe**	−2.424	−2.304	−2.467																	
**Ile**	−2.41	−2.286	−2.53	−2.691																
**Leu**	−2.343	−2.208	−2.491	−2.647	−2.501															
**Val**	−2.258	−2.079	−2.391	−2.568	−2.447	−2.385														
**Trp**	−2.08	−2.09	−2.286	−2.303	−2.222	−2.097	−1.867													
**Tyr**	−1.892	−1.834	−1.963	−1.998	−1.919	−1.79	−1.834	−1.335												
**Ala**	−1.7	−1.517	−1.75	−1.872	−1.728	−1.731	−1.565	−1.318	−1.119											
**Gly**	−1.101	−0.897	−1.034	−0.885	−0.767	−0.756	−1.142	−0.818	−0.29	0.219										

*Thr *	−1.243	−0.999	−1.237	−1.36	−1.202	−1.24	−1.077	−0.892	−0.717	−0.311	−0.617									
*Ser *	−1.306	−0.893	−1.178	−1.037	−0.959	−0.933	−1.145	−0.859	−0.607	−0.261	−0.548	−0.519								
*Gln *	−0.835	−0.72	−0.807	−0.778	−0.729	−0.642	−0.997	−0.687	−0.323	0.033	−0.342	−0.26	0.054							
*Asn *	−0.788	−0.658	−0.79	−0.669	−0.524	−0.673	−0.884	−0.67	−0.371	−0.23	−0.463	−0.423	−0.253	−0.367						
*Glu *	−0.179	−0.209	−0.419	−0.439	−0.366	−0.335	−0.624	−0.453	−0.039	0.443	−0.192	−0.161	0.179	0.16	0.933					
*Asp *	−0.616	−0.409	−0.482	−0.402	−0.291	−0.298	−0.613	−0.631	−0.235	−0.097	−0.382	−0.521	0.022	−0.344	0.634	0.179				
*His *	−1.499	−1.252	−1.33	−1.234	−1.176	−1.118	−1.383	−1.222	−0.646	−0.325	−0.72	−0.639	−0.29	−0.455	−0.324	−0.664	−1.078			
*Arg *	−0.771	−0.611	−0.805	−0.854	−0.758	−0.664	−0.912	−0.745	−0.327	−0.05	−0.247	−0.264	−0.042	−0.114	−0.374	−0.584	−0.307	0.2		
*Lys *	−0.112	−0.146	−0.27	−0.253	−0.222	−0.2	−0.391	−0.349	0.196	0.589	0.155	0.223	0.334	0.271	−0.057	−0.176	0.388	0.815	1.339	

** Pro**	−1.196	−0.788	−1.076	−0.991	−0.771	−0.886	−1.278	−1.067	−0.374	−0.042	−0.222	−0.199	−0.035	−0.018	0.257	0.189	−0.346	−0.023	0.661	0.129

	* Cys *	** Met**	** Phe**	** Ile**	** Leu**	** Val**	** Trp**	** Tyr**	** Ala**	** Gly**	* Thr *	* Ser *	* Gln *	* Asn *	* Glu *	* Asp *	* His *	* Arg *	* Lys *	** Pro**

**Table 3:** Combination of SS-Tabu variations amongst different threads.

Combinations	HP guide SS-Tabu	BM guide SS-Tabu
1 (PSSB4H0)	0 thread	4 threads
2 (PSSB3H1)	1 thread	3 threads
3 (PSSB2H2)	2 threads	2 threads
4 (PSSB1H3)	3 threads	1 thread
5 (PSSB0H4)	4 threads	0 thread

**Table 4:** For 9 medium sized proteins, the three different sets of excremental data—(i) our parallel local search framework (PSS), (ii) the tabu guided spiral search ( SS-Tabu ), and (iii) the genetic algorithms (GA^+^). The RI Columns present the relative improvements of parallel local search over the single-thread local search and the genetic algorithm. The RI is calculated on the average energy values.

			Our approach		The current state-of-the-art approaches					
			(Four threads)		(Single thread)					
Protein Info.			0.5 hrs × 4 = 2 hrs		2 hrs × 1 = 2 hrs					
			PSS		SS-Tabu [9]			GA^+^ [8]		
Seq	Size	LBFE	Best	Avg (*E*_*t*_)	Best	Avg (*E*_*r*_)	RI	Best	Avg (*E*_*r*_)	RI
F90_1	90	−168	**−168**	−166	−168	**−167**	**0**%	−168	−166	**0**%
F90_2	90	−168	**−168**	**−166**	−167	−164	**50**%	−168	−165	**33%**
F90_3	90	−167	**−167**	**−165**	−167	−165	0%	−167	−164	**33**%
F90_4	90	−168	**−168**	**−166**	−168	−165	33%	−168	−165	**33%**
F90_5	90	−167	**−167**	−165	−167	−165	0%	−167	**−166**	0%

S1	135	−357	**−355**	**−350**	−355	−347	30%	−355	−348	22%
S2	151	−360	**−356**	**−351**	−354	−347	31%	−356	−349	18%
S3	162	−367	**−360**	**−354**	−359	−350	26%	−361	−349	28%
S4	164	−370	**−364**	**−358**	−358	−350	40%	−364	−352	**33%**

**Table 5:** For 12 large sized proteins, the three different sets of excremental data—(i) our parallel local search framework (PSS), (ii) the tabu guided spiral search (SS-Tabu), and (iii) the genetic algorithms (GA^+^). The RI Columns present the relative improvements of parallel local search over the single-thread local search and the genetic algorithm. The RI is calculated on the average energy values.

			Our approach		The current state-of-the-art approaches					
			(Four threads)		(Single thread)					
Protein Info.			1.25 hrs × 4 = 5 hrs		5 hrs × 1 = 5 hrs					
			PSS		SS-Tabu [9]			GA^+^ [8]		
Seq	Size	LBFE	Best	Avg (*E*_*t*_)	Best	Avg (*E*_*r*_)	RI	Best	Avg (*E*_*r*_)	RI
F180_1	180	−378	**−359**	**−344**	−357	−340	11%	−351	−341	8%
F180_2	180	−381	**−364**	**−352**	−359	−345	19%	−362	−346	17%
F180_3	180	−378	**−368**	**−356**	−362	−353	12%	−361	−350	21%

R1	200	−384	**−366**	**−353**	−359	−345	21%	−355	−346	18%
R2	200	−383	**−368**	**−355**	−358	−346	24%	−360	−346	**24%**
R3	200	−385	**−369**	**−353**	−365	−345	20%	−363	−344	22%

3mse	179	−323	**−296**	**−285**	−289	−280	12%	−290	−279	14%
3mr7	189	−355	**−332 **	**−319**	−328	−313	14%	−328	−316	8%
3mqz	215	−474	**−430**	**−414**	−420	−402	17%	−427	−410	6%
3no6	229	−455	**−429**	**−407**	−411	−391	**25%**	−420	−400	13%
3no3	258	−494	**−422**	**−404**	−412	−393	**11%**	−421	−402	**2%**
3on7	279	n/a	**−516**	**−500**	−512	−485	n/a	−515	−485	n/a

**Table 6:** The benchmark proteins used in our experiments.

ID	Length	Sequence
* 4RXN *	54	MKKYTCTVCGYIYNPEDGDPDNGVNPGTDFKDIPDDWVCPLCGVGKDQFEEVEE
* 1ENH *	54	RPRTAFSSEQLARLKREFNENRYLTERRRQQLSSELGLNEAQIKIWFQNKRAKI
* 4PTI *	58	RPDFCLEPPYTGPCKARIIRYFYNAKAGLCQTFVYGGCRAKRNNFKSAEDCMRTCGGA
* 2IGD *	61	MTPAVTTYKLVINGKTLKGETTTKAVDAETAEKAFKQYANDNGVDGVWTYDDATKTFTVTE
* 1YPA *	64	MKTEWPELVGKAVAAAKKVILQDKPEAQIIVLPVGTIVTMEYRIDRVRLFVDKLDNIAQVPRVG
* 1R69 *	69	SISSRVKSKRIQLGLNQAELAQKVGTTQQSIEQLENGKTKRPRFLPELASALGVSVDWLLNGTSDSNVR
* 1CTF *	74	AAEEKTEFDVILKAAGANKVAVIKAVRGATGLGLKEAKDLVESAPAALKEGVSKDDAEALKKALEEAGAEVEVK

* 3MX7 *	90	MTDLVAVWDVALSDGVHKIEFEHGTTSGKRVVYVDGKEEIRKEWMFKLVGKETFYVGAAKTKATINIDAISGFA
		YEYTLEINGKSLKKYM
* 3NBM *	108	SNASKELKVLVLCAGSGTSAQLANAINEGANLTEVRVIANSGAYGAHYDIMGVYDLIILAPQVRSYYREMKVDAE
		RLGIQIVATRGMEYIHLTKSPSKALQFVLEHYQ
* 3MQO *	120	PAIDYKTAFHLAPIGLVLSRDRVIEDCNDELAAIFRCARADLIGRSFEVLYPSSDEFERIGERISPVMIAHGSYADDR
		IMKRAGGELFWCHVTGRALDRTAPLAAGVWTFEDLSATRRVA
* 3MRO *	142	SNALSASEERFQLAVSGASAGLWDWNPKTGAMYLSPHFKKIMGYEDHELPDEITGHRESIHPDDRARVLAALK
		AHLEHRDTYDVEYRVRTRSGDFRWIQSRGQALWNSAGEPYRMVGWIMDVTDRKRDEDALRVSREELRRL
* 3PNX *	160	GMENKKMNLLLFSGDYDKALASLIIANAAREMEIEVTIFCAFWGLLLLRDPEKASQEDKSLYEQAFSSLTPREAE
		ELPLSKMNLGGIGKKMLLEMMKEEKAPKLSDLLSGARKKEVKFYACQLSVEIMGFKKEELFPEVQIMDVKEYLK
		NALESDLQLFI

**Table 7:** The best and average contact energies obtained from 8 different approaches using Berrera et al. [25] 20 × 20 energy matrix. Rowwise bold-faced values are the winners for the corresponding proteins amongst the variants of spiral search (both single and parallel frameworks) and bold-italic-faced values are the winners for the corresponding proteins amongst all 8 approaches. For both energy and RMSD values, the lower the better.

Comparing all-atomic interaction energy and RMSD values																		
			State-of-the-art		Spiral search		Parallel spiral search (PSS) variants on energy model mixing										State-of-the-art	
			CPU time 1 hr		CPU time 1 hr		CPU time 1 hr (4-threads × 15 minutes))										CPU time 1 hr	
Protein details			1 × br-thread		1 × br-thread		4 × br-threads		3 × br-threads		2 × br-threads		1 × br-thread		0 × br-thread		br and hp based	
			0 × hp-thread		0 × hp-thread		0 × hp-thread		1 × hp-thread		2 × hp-threads		3 × hp-threads		4 × hp-threads		single threaded	
			LS-Tabu [10]		SS-Tabu		PSSB4H0		PSSB3H1		PSSB2H2		PSSB1H3		PSSB0H4		GA^+^ [11]	
Seq.	Size	H	Energy	RMSD	Energy	RMSD	Energy	RMSD	Energy	RMSD	Energy	RMSD	Energy	RMSD	Energy	RMSD	Energy	RMSD
*4RXN *	54	27	*−156.32 *	* 6.29 *	−150.11	6.00	−142.22	5.23	−154.47	5.21	−156.94	** 5.11**	−157	5.19	**−157.25**	5.17	***−162.72***	* 5.41 *
*1ENH *	54	19	*−146.69 *	* 6.61 *	−143.01	5.88	−129.23	5.11	−146.88	5.09	−147.76	5.02	**−148.58**	4.93	−148.39	** 4.90**	***−151.65***	* 5.22 *
*4PTI *	58	32	*−198.42 *	* 7.07 *	−190.77	6.99	−175.52	6.41	−196.05	6.27	−197.33	6.38	**−198.42**	6.38	−198.3	** 6.37**	***−204.56***	* 6.46 *
*2IGD *	61	25	*−174.19 *	* 9.33 *	−163.87	8.50	−151.09	** 7.26**	−171.74	** 7.26**	−172.83	7.28	−173.89	7.33	**−174.02**	7.43	***−176.83***	* 7.81 *
*1YPA *	64	38	*−239.98 *	* 7.53 *	−236.10	6.86	−214.60	6.00	−245.19	6.05	−248.43	5.93	−247.35	** 5.77**	**−248.54**	5.86	***−253.09***	* 6.29 *
*1R69 *	69	30	*−204.17 *	* 6.47 *	−191.14	5.65	−175.61	5.14	−203.22	4.92	−204.81	4.85	−205.88	4.88	**−207.06**	** 4.78**	***−208.79***	*** 5.17***
*1CTF *	74	42	*−213.81 *	* 7.23 *	−197.85	5.63	−179.18	5.23	−218.38	5.21	−220.1	5.06	**−222.23**	** 5.02**	−221.67	5.06	***−225.42***	* 5.28 *

* 3MX7 *	90	44	*−311.56 *	* 8.18 *	−300.89	8.62	−257.49	7.87	−321.94	8.00	−324.09	7.8	**−326.23**	7.70	−325.55	** 7.64**	*−325.45 *	* 7.94 *
*3NBM *	108	56	*−401.99 *	* 8.58 *	−380.12	6.95	−329.7	6.88	−409.5	6.12	−406.74	6.06	**−412.96**	6.06	−411.18	** 6.00**	***−419.25***	* 6.46 *
*3MQO *	120	68	*−455.27 *	* 8.86 *	−422.4	7.52	−336.74	7.39	−461.38	6.98	−465.02	6.83	**−469.27**	6.77	−467.38	** 6.67**	***−472.78***	* 6.84 *
*3MRO *	142	63	* 430.29 *	* 10.02 *	−397.14	9.61	−313.85	8.74	−445.23	8.11	−450.68	7.93	−448.59	7.85	**−452.04**	** 7.71**	*−447.77 *	* 8.72 *
*3PNX *	160	84	*−571.13 *	* 9.38 *	−502.29	9.55	−383.49	9.05	−586.68	8.73	−593.85	** 8.38**	−595.99	8.45	−**600.18**	8.39	*−592.25 *	* 8.51 *
